# 3D radiomics profiling of thyroid tumors using micro-CT

**DOI:** 10.1038/s41598-026-57746-1

**Published:** 2026-06-17

**Authors:** Kiarash Tajbakhsh, Olga Stanowska, Marija Buljan, Antonia Neels, Aurel Perren, Robert Zboray

**Affiliations:** 1 Center for X-ray Analytics, Swiss Federal Laboratories for Materials Science and Technology (Empa), Dübendorf, 8600 Switzerland; 2 Department of Chemistry, University of Fribourg, Fribourg, 1700 Switzerland; 3 Institute of Tissue Medicine and Pathology, University of Bern, Bern, 3008 Switzerland; 4 Nanomaterials in Health, Swiss Federal Laboratories for Materials Science and Technology (Empa), St. Gallen, 9014 Switzerland; 5 Swiss Institute of Bioinformatics (SIB), Lausanne, 1015 Switzerland; 6 ARTORG Center, University of Bern, Bern, 3010 Switzerland

**Keywords:** Radiomics, Thyroid neoplasms, Micro-CT, BRAF V600E, TERT, Biomarkers, Cancer, Computational biology and bioinformatics, Oncology

## Abstract

Tumor heterogeneity plays a central role in treatment resistance, disease progression, and diagnostic uncertainty. However, it may be overlooked by traditional 2D histology. Accurate 3D assessment of tumor microarchitecture is therefore important for capturing its spatial complexity. Micro-CT, a well-established imaging modality now emerging for high-resolution 3D virtual histology of soft tissues, provides a promising alternative. Combined with radiomics, this technique enables interpretable, quantitative characterization of tumor biology beyond visual inspection. In this study, we analyzed radiomics signatures of a large cohort of thyroid tumors (418 patients) using micro-CT imaging of tissue microarrays. We achieved robust classification of (i) neoplastic versus non-neoplastic thyroid tissues, (ii) papillary thyroid carcinoma versus follicular thyroid neoplasm, and (iii) BRAF V600E mutation status. Shapley additive explanations were used to reveal key visual traits driving these classification decisions. Exploratory analysis in a limited TERT cohort (8 mutated vs 103 wild-type) identified prospective radiomics patterns associated with TERT promoter mutations, suggesting potential surrogate imaging biomarkers that warrant further investigation. Micro-CT radiomics shows promise as a complementary tool for diagnostic classification in thyroid cancer and offers a platform for quantitative 3D tissue characterization pending broader validation.

## Introduction

Tumors are dynamic entities that evolve following a Darwinian model of clonal selection and adaptation. Environmental and therapeutic pressures can convert an initially monoclonal lesion into an ecosystem whose subclones differ in molecular profile, morphology, and stromal context. Some of these subclones can develop drug resistance, replicative immortality, and cooperative metastasis^1,2^. Thyroid cancer is no exception, yet its malignancies are still classified primarily by conventional histology, which may overlook this spatial heterogeneity.

Five major histotypes of thyroid cancer are recognised: papillary (PTC), follicular (FTC), poorly differentiated (PDTC), medullary and anaplastic carcinoma, with PTC and FTC accounting for 80% and 15% of cases, respectively^3–5^. Prognostic assessment in PTC primarily relies on architectural and cytological features, whereas the diagnosis and risk stratification of FTC depend on the presence and extent of capsular and vascular invasion, while mitotic activity and necrosis are important prognostic indicators in both subtypes.

Borderline tumors blur these criteria: the follicular variant of PTC (FVPTC) shows both PTC- and FTC-like characteristics and low inter-observer agreement^6,7^. The diagnosis of follicular carcinoma is similarly contentious, as what constitutes genuine vascular invasion remains subject to interobserver discordance^8^. Beyond this interpretive ambiguity, the focal nature of capsular and vascular invasion makes conventional 2D histology vulnerable to sampling bias, increasing the risk of both over- and under-diagnosis^9^.

Molecular profiling is becoming increasingly important in the diagnosis and prognosis of thyroid carcinomas^10^. In PTC, co-occurrence of BRAF V600E and TERT-promoter mutations identifies tumors with aggressive trajectories; in FTC, TERT-promoter mutations and RAS alterations likewise portend poorer outcomes^11–14^. Crucially, these driver events are themselves heterogeneous: TERT mutations can be patchy within FTC^15^, while multifocal or polyclonal PTCs may harbor discordant BRAF and RAS genotypes^16–18^. Such evolution-driven intratumoral heterogeneity complicates diagnosis and risk stratification.

Immunohistochemistry is performed on individual 2D tissue sections, each sampling only a small fraction of the tumor volume and therefore susceptible to missing spatially distinct subclonal populations or focal invasion events. Three-dimensional imaging of the tissue embedded in FFPE blocks addresses this limitation by capturing the full spatial continuity of microarchitectural features within the sampled volume. Molecular assays lose spatial information in a different way: PCR-based sequencing homogenises milligrams of tissue into a single bulk measurement that raises sampling questions^19^.

If tumor phenotype is correlated with its molecular characteristics, the phenotype may serve as a proxy for the underlying genotype. This would allow molecular characteristics to be inferred from imaging of tissue embedded in formalin-fixed paraffin-embedded (FFPE) blocks.

Meanwhile, two imaging modalities have emerged to probe soft tissues in three dimensions^20^. Fluorescence-based techniques, such as light-sheet microscopy, operate at optical wavelengths and provide cellular resolution with cell-specific contrast^21^. However, these methods typically require extensive non-reversible chemical sample preparation, i. e. tissue clearing and fluorescent staining^22^. By contrast, micro-CT can accommodate larger samples, is fast, and does not necessitate additional chemical treatments, enabling non-destructive visualisation of the tumor micro-architecture at high fidelity, though it lacks cell-specific contrast and resolution^23^.

Micro-CT has already demonstrated clinical benefits, supporting histology of FTCs by providing 3D insight into vascular and capsular invasion^9,24,25^, propagating 2D spatial transcriptomics to 3D volumes^26,27^, and prostate cancer prognosis^28^. Micro-CT does not require any sample preprocessing other than FFPE block preparation. High-quality scans of FFPE tissue blocks can be acquired within two hours^23^, a negligible and fitting addition to the current clinical workflow turnaround of a few days.

The texture patterns captured in micro-CT images may reflect underlying biological processes and can be computationally mined to infer molecular profiles and stratify risk^29^. Classical radiomics, built on hundreds of hand-crafted intensity, shape, and texture descriptors, has already connected such imaging signatures to actionable genetic alterations while remaining transparent and interpretable^30^. Deep-learning pipelines learn richer, task-specific representations, but they typically require large, well-annotated datasets and their latent features can be opaque to pathologists^31^.

Radiomics has been applied across a wide range of tasks and modalities, including prediction of lymph node metastasis in PTC from ultrasound, CT, and MRI^32–34^, detection of TERT and IDH mutations in brain tumors^35,36^, EGFR subtyping in lung adenocarcinoma^37^, and KRAS prediction in colorectal cancer^38^.

We applied micro-CT radiomics across several clinically relevant classification tasks in a large cohort of thyroid tumors. First, we distinguished neoplastic from non-neoplastic thyroid parenchyma. Subsequently, we classified papillary thyroid carcinoma (PTC) against follicular thyroid neoplasms (FTN), encompassing both follicular thyroid carcinomas and adenomas. We also predicted BRAF V600E mutation status. For the tumor type and BRAF classification tasks, Shapley additive explanations (SHAP)^39^ were used to reveal the key textural patterns driving these classifications. We also observed preliminary associations between specific radiomics features and TERT promoter mutations. RAS Q61R point mutation and relapse were also studied; however, no reliable classification could be established based on the current study design and modeling conditions. The classifiers were trained on ngTMA punches, which provide well-labeled, high-throughput training data under controlled conditions; scanning whole tumor blocks at 5 µm voxel size for a cohort of this scale would be impractically time-intensive. In the intended downstream application, a whole micro-CT tumor volume can be tiled into patches matching the punch size and classified independently, producing a spatially resolved 3D map of tissue type or mutation probability across the entire tumor.

## Results and discussion

### Tissue type

**Fig. 1:**
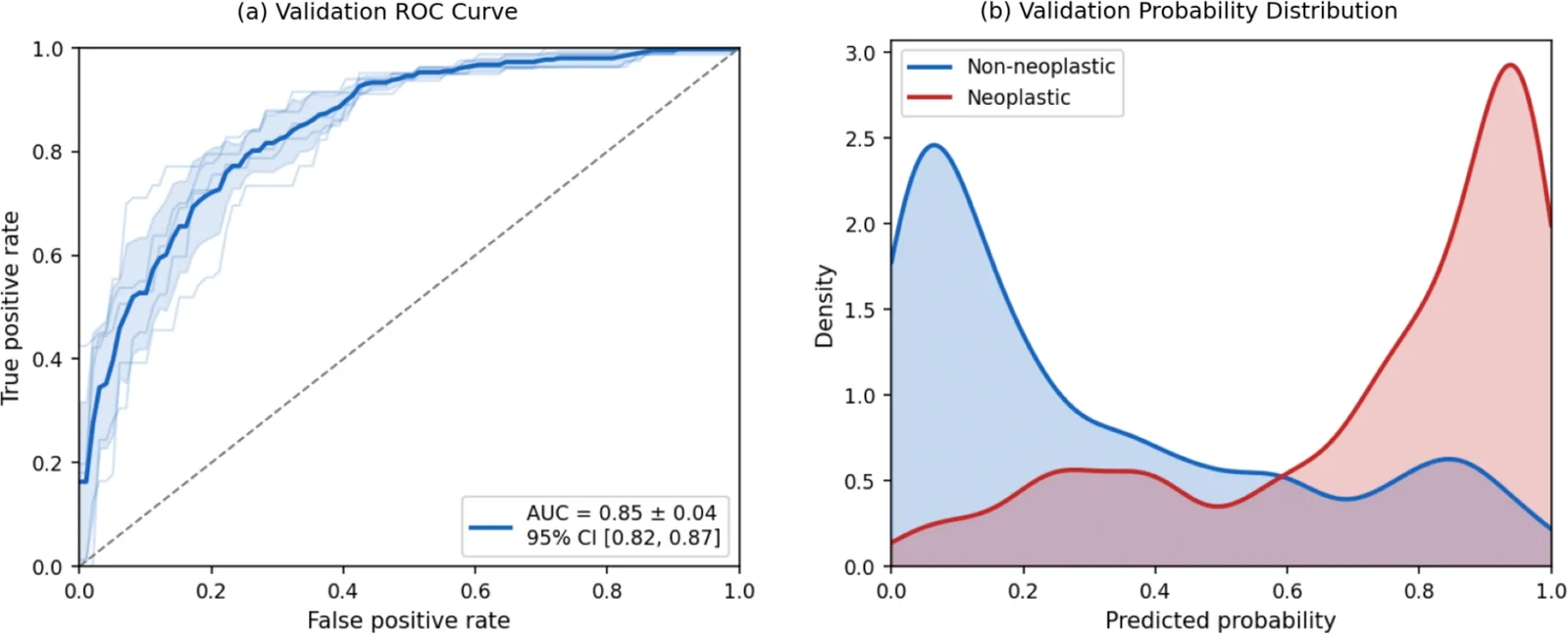
Tissue type classification, distinguishing non-neoplastic (n=336) from neoplastic (n=405) thyroid tissue. (**a**) Validation ROC curve across 5-fold cross-validation; AUC = 0.85, 95% CI [0.82, 0.87]; AUPRC = 0.85, 95% CI [0.80, 0.89]. (**b**) Predicted probability distributions for non-neoplastic and neoplastic tissue across validation folds.

We first evaluated the model’s ability to distinguish non-neoplastic (n=336) from neoplastic (n=405) thyroid tissue. Receiver operating characteristic (ROC) analysis across the validation folds in a 5-fold cross-validation yielded an area under the curve (AUC) of 0.85 ± 0.04 Fig. 1(a), confirming robust generalisation. The predicted probability distributions Fig. 1(b) exhibit distinct bimodality, with minor overlap between classes due to biological gray zones or a mixed presence of neoplastic and non-neoplastic tissue in some tissues. A Jensen–Shannon divergence (JSD) of 0.325 confirms strong distributional separation between the two classes. Additional performance metrics and dataset details are provided in table S2. The strong separation between classes is expected: non-neoplastic thyroid parenchyma exhibits a regular follicular architecture with uniformly distributed, colloid-filled follicles that produce a periodic, homogeneous texture in micro-CT. Neoplastic tissue disrupts this regularity through altered follicle size distribution, increased cellularity, and variable colloid content, resulting in a measurably more heterogeneous textural profile. Together, these results demonstrate that our radiomics model reliably identifies the textural signatures that differentiate neoplastic from non-neoplastic tissue.

### Tumor type

The classifier achieved strong performance for FTN (n=116) and PTC (n=97), which together account for up to 95% of thyroid neoplasms. The dataset details of the classification metrics are given in S2. It is important to note that FTN includes both follicular thyroid adenomas (FA) and follicular thyroid carcinomas (FTC). The histopathological distinction between FA and FTC is based on the presence of capsular and vascular invasion, not on textural features. Since both FA and FTC exhibit the same follicular growth pattern, textural differences between FA and FTC are not expected by default, though they cannot be entirely excluded. Therefore, this classifier captures radiomics features that differentiate PTC from follicular-patterned lesions (regardless of adenoma or carcinoma status).

**Fig. 2:**
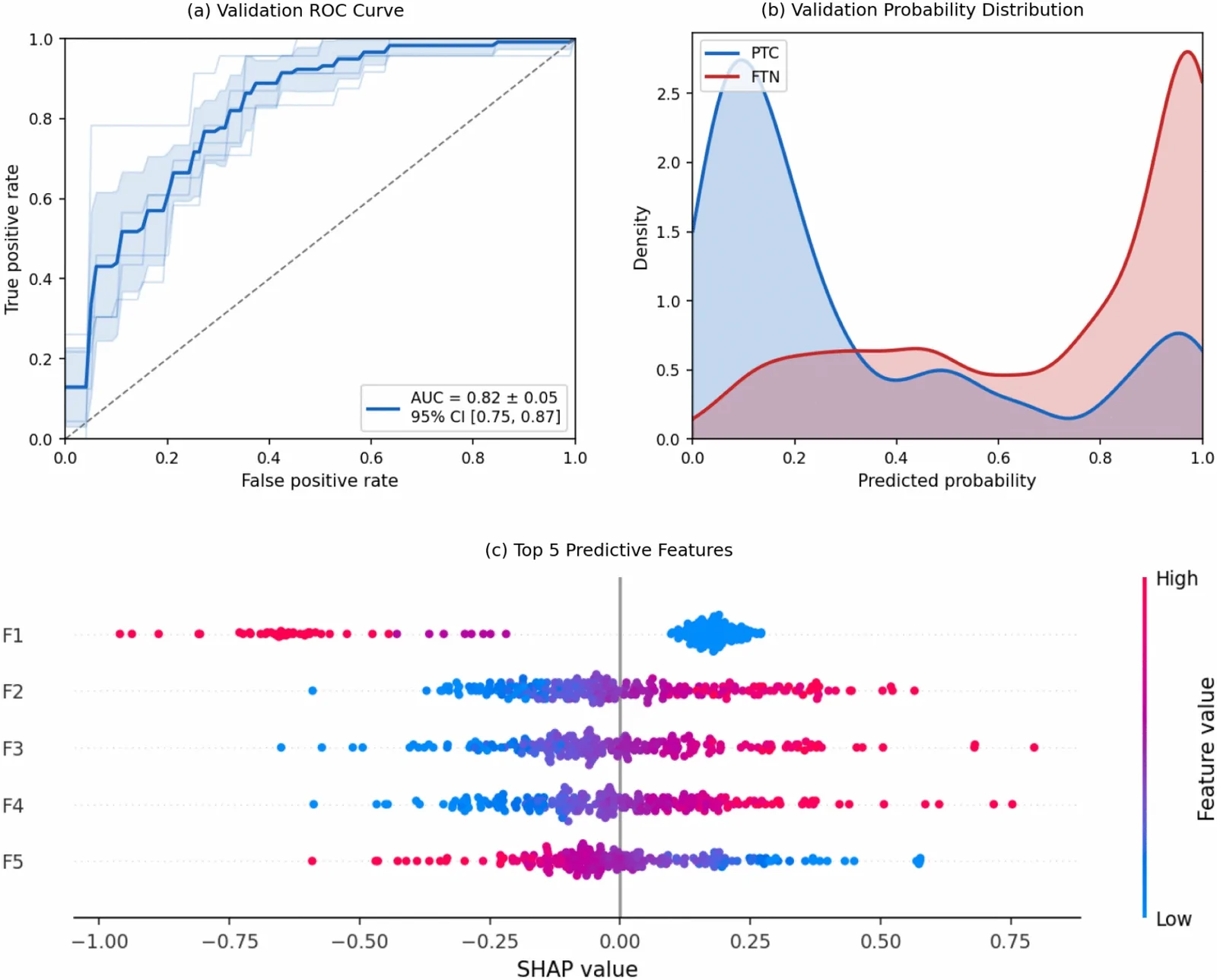
Classification of papillary thyroid carcinoma (PTC, n=97) versus follicular thyroid neoplasm (FTN, n=116). (**a**) Validation ROC curve across 5-fold cross-validation; AUC = 0.82, 95% CI [0.75, 0.87]; AUPRC = 0.82, 95% CI [0.72, 0.88]. (**b**) Predicted probability distributions for PTC and FTN across validation folds. (**c**) Top 5 predictive radiomics features by mean absolute SHAP value; color indicates feature value (blue: low, red: high). F1: wavelet-LLH first-order Median; F2: wavelet-LHH GLSZM Zone Entropy; F3: wavelet-LLH GLCM Sum Average; F4: LoG-10µm GLRLM Long Run Low Gray Level Emphasis; F5: LoG-10µm GLRLM High Gray Level Run Emphasis.

The classifier achieved a validation AUC of 0.82 ± 0.05 in Fig. 2(a), indicating robust generalizability across validation folds. Furthermore, the model’s probability distribution in Fig. 2(b) shows a distinct bimodal pattern, supporting the model’s ability to differentiate the two tumor groups despite some overlap. A JSD of 0.298 quantifies the distributional separation between PTC and FTN predicted probabilities.

SHAP analysis in Fig. 2(c) identifies the five most informative features for the PTC versus FTN task; please note the wavelet directional channels carry no orientation-specific meaning as the punches do not possess directional information (see Fig. S4). Two features push predictions toward PTC: a high median of the wavelet detail band (F1), indicating fine-scale intensity fluctuations uniformly elevated across the punch, and a high LoG-10 µm GLRLM High Gray Level Run Emphasis (F5), indicating frequent runs of bright voxels at the 10 µm scale. Together these describe densely and consistently textured tissue with pervasive bright micro-structures, consistent with the packed architecture of papillary growth. The remaining three features push predictions toward FTN: a high GLSZM Zone Entropy (F2), reflecting greater variability in the size and intensity of homogeneous zones; a high GLCM Sum Average (F3), reflecting brighter co-occurring voxel pairs; and a high LoG-10 µm GLRLM Long Run Low Gray Level Emphasis (F4), reflecting extended contiguous runs of low-intensity voxels. Together these describe a patchwork of variably sized homogeneous regions alternating between bright and dark, consistent with discrete follicles of differing diameter whose iodine-rich colloid forms bright lumens set within a darker follicular environment. These inferred image patterns are in line with the PTC (**) and FTC (*) punches shown in Fig. 4(b).

### Follicular variant of papillary thyroid carcinomas

Clinically, FVPTC is recognised as an intermediate entity between PTC and FTC with nuclear features of PTC and invasive growth pattern of FTC^40^.

To quantify the distributional affinity of FVPTC to each reference class, we applied the trained PTC vs. FTN classifier to the *n=47* FVPTC samples as an out-of-distribution test. The mean predicted probability of PTC across FVPTC samples was 0.52 (median 0.58, std 0.29), placing FVPTC near the decision boundary but on the PTC side. Jensen–Shannon divergence confirmed this directionality: JSD(FVPTC, PTC) = 0.227 was lower than JSD(FVPTC, FTN) = 0.324, indicating that the FVPTC probability distribution is more similar to PTC than to FTN (see Fig. S1).

Together, these results confirm that FVPTC occupies an intermediate position in the radiomics feature space, with a quantifiable lean towards PTC. This textural affinity does not capture nuclear features, as these are not resolved at this imaging resolution. The distributional overlap is consistent with the intrinsic diagnostic ambiguity of FVPTC, which is also associated with high intra- and inter-observer variability^6^.

### BRAF V600E status

The BRAF gene encodes a kinase involved in the Mitogen-Activated Protein Kinase signaling pathway, which regulates cell proliferation and survival. Mutations in BRAF, particularly V600E, result in constitutive pathway activation and are frequently implicated in tumorigenesis. BRAF mutations are recently reported to correlate with increased resistance to radioiodine therapy^41^, but potentially increased response to tyrosine kinase inhibitors^42^. BRAF mutation is highly prevalent (89%) in aggressive classical PTC^43^, making it a key prognostic marker. In contrast, BRAF mutations are absent in FTN^12^, therefore, FTNs were excluded from the dataset of this classification task. The dataset and details of the classification metrics are given in table S2. While BRAF mutations are generally homogeneous^44^, intratumoral heterogeneity, particularly of BRAF status in multifocal^16^ and polyclonal PTCs, has been reported^18^, highlighting the potential value of 3D imaging modalities like micro-CT for mutation status classification.

**Fig. 3:**
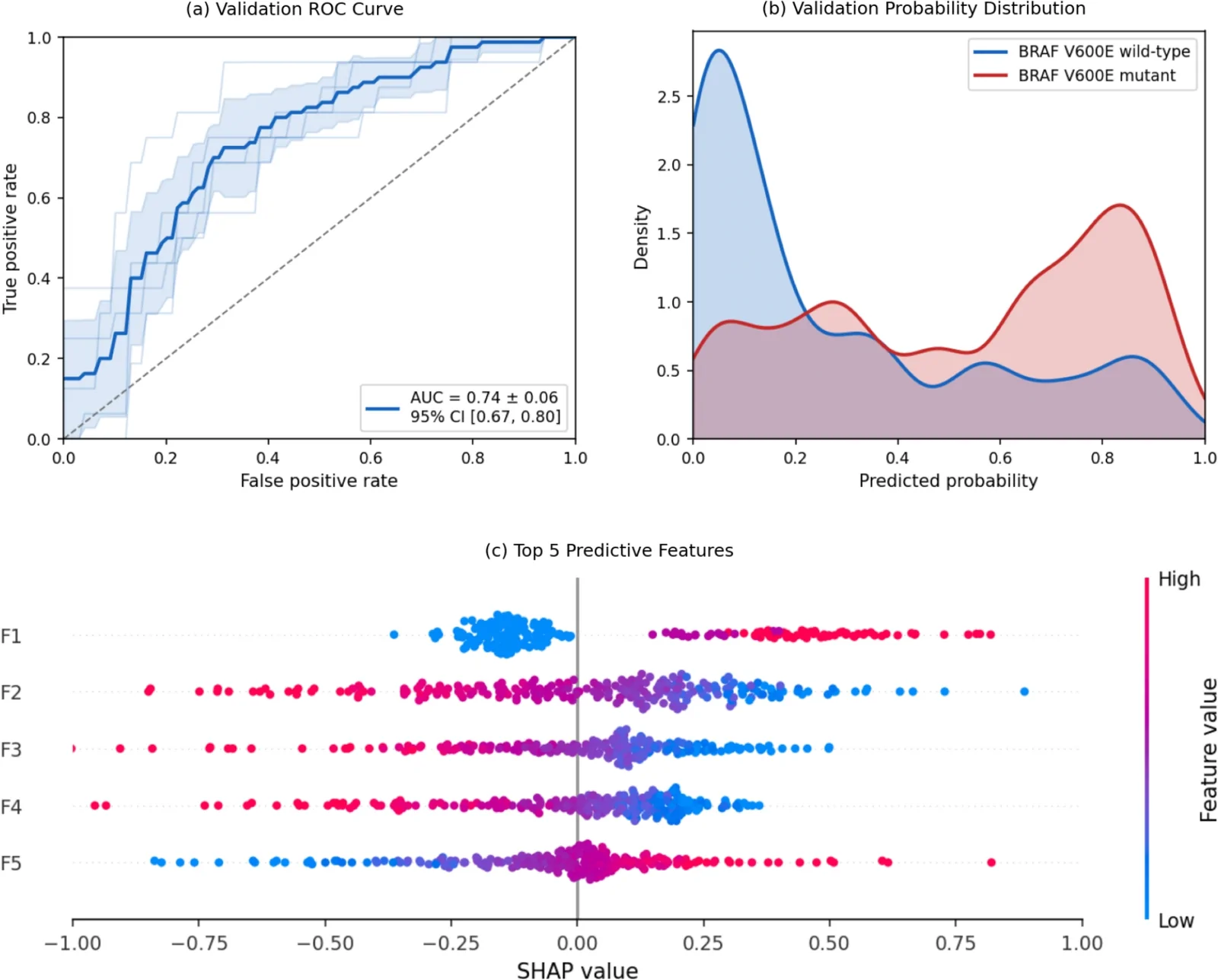
BRAF V600E mutation status classification, distinguishing BRAF wild-type (n=159) from BRAF mutant (n=80) tumors. (**a**) Validation ROC curve across 5-fold cross-validation; AUC = 0.74, 95% CI [0.67, 0.80]; AUPRC = 0.59, 95% CI [0.44, 0.66]. (**b**) Predicted probability distributions for BRAF wild-type and mutant across validation folds. (**c**) Top 5 predictive radiomics features by mean absolute SHAP value; color indicates feature value (blue: low, red: high). F1: wavelet-LLH first-order Median; F2: LoG-10µm GLSZM Gray Level Non-Uniformity; F3: LoG-10µm GLRLM Long Run Low Gray Level Emphasis; F4: wavelet-HLL GLSZM Gray Level Non-Uniformity Normalized; F5: LoG-10µm GLRLM High Gray Level Run Emphasis.

The classification performance of the proposed model for predicting BRAF V600E mutation status (determined by immunohistochemistry with the VE1 clone, see section “Immunohistochemistry”) is summarized in Fig. 3. The dataset comprises BRAF V600E-positive (n=80) and BRAF V600E-negative (n=159) tumors. ROC analysis Fig. 3(a) yields a mean AUC of 0.74 ± 0.06 in 5-fold cross validation, suggesting that the model achieves moderate discrimination between two classes. The relatively small standard deviation reflects consistent performance across validation folds. The predicted probability distributions Fig. 3(b) show a reasonable separation between classes. A JSD of 0.222 quantifies this separation, reflecting the more limited distributional distance consistent with the moderate AUC and class imbalance. The model could in principle be extended to screen the full tumor volume for BRAF V600E status and provide a spatially resolved map of intratumoral heterogeneity.

Figure 3(c) shows the SHAP summary for the classification of BRAF V600E mutation status. The five most informative features split into two opposing groups. Two favor the mutant class: a high median of the wavelet detail band (F1) and a high LoG-10 µm GLRLM High Gray Level Run Emphasis (F5), both indicating elevated and frequent high-intensity fine-scale texture at the 10 µm level. Three favor the wild-type class: a high LoG-10 µm GLSZM Gray Level Non-Uniformity (F2) and its normalized counterpart in the wavelet detail band (F4), both indicating that zone intensities are concentrated in a few dominant gray-level values, and a high LoG-10 µm GLRLM Long Run Low Gray Level Emphasis (F3), indicating extended contiguous runs of low-intensity voxels. Read together, the SHAP profile describes mutant tumors as having pervasive, intense fine-scale micro-structural activity, while wild-type tumors present a more uniform texture with larger low-intensity regions. An example of a BRAF V600E-positive tumor is shown in Fig. S2(a) with higher textural activity and heterogeneity consistent with the SHAP analysis in section "BRAF V600E status". The classifier predicts 84% and 36% BRAF V600E mutation probability for Fig. S2(a), and (b), respectively. Below each punch, the immunohistochemistry image for BRAF V600E is shown.

### TERT status

Since their discovery, the TERT promoter mutations have emerged as key prognostic genetic alterations in thyroid cancer. These promoter mutations create de novo E26 transformation-specific (ETS) transcription factor binding motifs, leading to increased TERT expression and cellular immortalization. The prevalence of TERT mutation in benign tumors is zero, while it increases to 11% in PTC, 17% in FTC, 43% in PDTC, and 40% in anaplastic thyroid carcinoma. Their enrichment in aggressive subtypes underscores their association with adverse clinical outcomes, including recurrence and mortality^45^. These findings position TERT promoter mutations as promising biomarkers for risk stratification and potential therapeutic targets in thyroid cancer. Motivated by the clinical significance of TERT mutations, we applied an exploratory radiomics analysis to the limited available dataset of 8 TERT-mutated and 103 wild-type tumors, with TERT promoter status determined by Sanger sequencing (see section "TERT mutational Analysis").

**Table 1:** Top 5 radiomics features ranked by raw p-value from the Mann–Whitney U test comparing TERT-mutated (n=8) and wild-type (n=103) tumors.

**Feature**	**U-statistic**	**p-value**	**Effect size (r)**
Wavelet-HLH first-order Median	254	0.058	0.38
Wavelet-HLH GLSZM Zone Percentage	569	0.074	−0.38
Wavelet-HLH GLRLM Long Run High Gray Level Emphasis	276	0.124	0.33
Wavelet-LLL first-order Kurtosis	291	0.173	0.29
Wavelet-LLL first-order Skewness	292	0.176	0.29

To better understand the underlying signal, we applied the non-parametric Mann–Whitney U test to each radiomics feature, comparing TERT-mutated and wild-type tumors. The top-ranked features by raw p-value are reported in table 1. None survive Benjamini–Hochberg FDR correction at q < 0.05 across 54 features tested. However, the top features show moderate effect sizes (|r| up to 0.38), suggesting that a genuine signal may be present but that the cohort is insufficiently powered to confirm it. Prospective studies with larger TERT-mutant cohorts are needed to validate these preliminary observations.

### RAS Q61R status

RAS mutations are among the most common genetic alterations in follicular-patterned thyroid tumors, occurring in follicular adenomas, follicular thyroid carcinomas, and the follicular variant of PTC. Unlike BRAF V600E, which is strongly associated with the classical PTC architecture, RAS mutations drive a follicular growth pattern and are generally associated with a less aggressive clinical course. The Q61R point mutation, detectable by the SP174 antibody, represents one variant within a broader family of RAS alterations spanning HRAS, KRAS, and NRAS^46^. Using a dataset of 308 wild-type and 46 RAS Q61R mutated tumors (RAS Q61R status determined by immunohistochemistry with the SP174 antibody, see section “Immunohistochemistry”), no robust radiomics signal was identified under the present sampling and modeling conditions (table S2). This finding is itself informative: it suggests that RAS Q61R mutation does not produce a distinct microstructural phenotype detectable. This is consistent with the fact that other RAS mutations (HRAS, KRAS, NRAS) can lead to similar morphologies and molecular alterations^47^, potentially diluting any mutation-specific signal. The imaging condition is not the limiting factor here; rather, the constraint is label specificity. The antibody detects only the Q61R variant, while other RAS mutations producing similar follicular morphologies are present in the wild-type group, obscuring any variant-specific microstructural signature. A sequencing-based labeling strategy capturing all RAS mutations would be a more appropriate approach for future studies.

### Relapse Risk

Relapse risk in thyroid cancer is determined by a combination of patient-level factors, clinical, pathological, and molecular characteristics that operate at the level of the whole tumor and its local spread, rather than at the microarchitectural scale captured by a single tissue core. Predicting relapse from a 600 µm punch therefore represents a fundamentally ill-posed task, as the relevant prognostic information is unlikely to be encoded within a single core at any resolution. The evaluation of the relapse prediction model using 128 disease-free survival and 95 relapsed cases yielded performance comparable to random classification (table S2), indicating no meaningful association between textural features and relapse risk. These results indicate that no robust radiomics signal for relapse was identified in this dataset under the present sampling and modeling conditions.

## Conclusion

Radiomics applied to 3D micro-CT images enables quantitative capture of complex textural patterns in tumor tissue. In this study, we demonstrated that radiomics analysis of 3D micro-CT images enables reliable classification of neoplastic versus non-neoplastic tissue, PTC versus FTN, and BRAF V600E mutation status. Exploratory analysis suggests that radiomics biomarkers for TERT promoter mutations may exist, though validation in larger cohorts is required. RAS Q61R and relapse prediction did not yield reliable classifiers, for reasons rooted in label specificity and sampling design rather than imaging limitations. Importantly, the 3D nature of the method enables characterization of intratumoral microarchitecture at the punch level. The micro-CT radiomics presented here represents a promising research platform for quantitative 3D characterization of thyroid tumor microarchitecture. Translating these findings into clinical practice will require external validation in independent cohorts and benchmarking against established clinical workflows.

## Methods

### Dataset

The next-generation tissue microarrays (ngTMA) represent a key enabler for scaling this technology^48^. By imaging multiple tissue cores under uniform conditions, ngTMAs reduce scan time, increase throughput, and ensure consistency in radiomics feature extraction, addressing a major bottleneck in the adoption of 3D imaging modalities for data-driven diagnostics tasks.

**Fig. 4:**
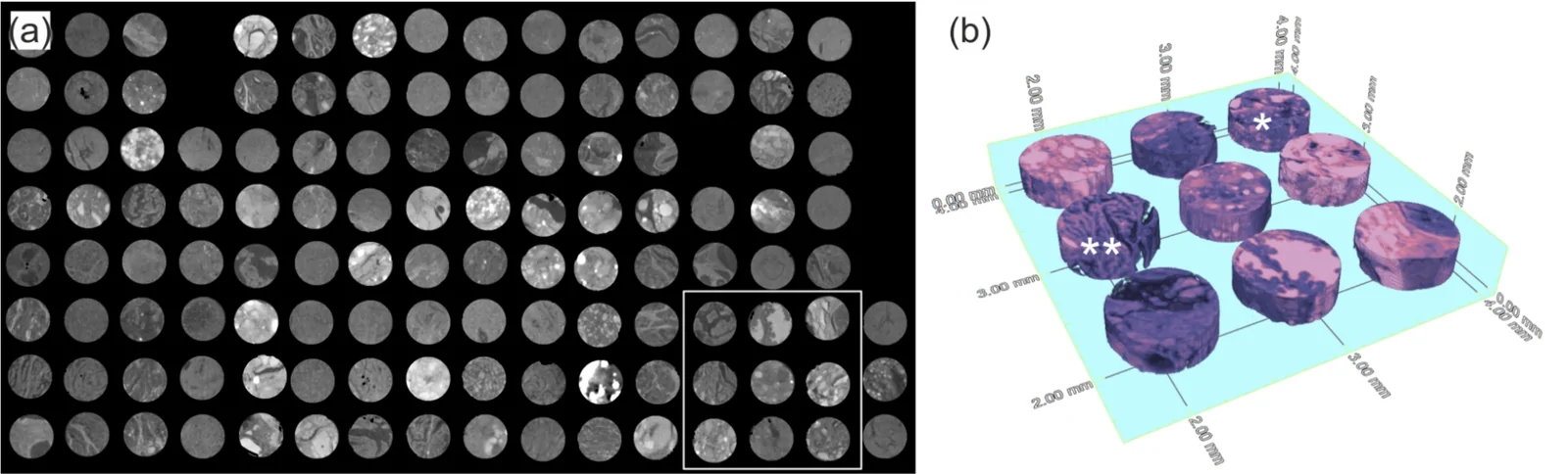
(**a**) A slice of an ngTMA batch with 120 punches, (**b**) A false color 3D rendering of a 3*×*3 grid of ngTMA area indicated by the white rectangle in (**a**). The punches annotated by * and ** are FTC and PTC, respectively.

Figure 4(b) illustrates a 3D rendering of a micro-CT scan depicting a 3x3 grid subvolume from a ngTMA, artificially colored to mimic hematoxylin and eosin staining. Each punch represents an individual specimen, with typical ngTMA blocks containing approximately 200 punches (see Fig. 4(a)). Our study analyzed four ngTMA blocks from two centers (University Hospital Zürich and University of Bern), resulting in a dataset comprising 336 non-neoplastic and 405 neoplastic tissue samples. Most patients provided paired punches: one from non-neoplastic peripheral tissue and one from neoplastic tissue. The overall cohort included 418 patients, though effective sample sizes varied according to specific classification tasks and the tumors data availability, as detailed in table S2. As each punch samples a 600 µm diameter core, it represents a limited spatial region of the tumor and may not fully capture intratumoral heterogeneity; this constitutes an inherent sampling limitation that should be considered when interpreting the radiomics features extracted from these cores.

### X-ray micro-CT

X-ray micro-CT was done using the EasyTom XL Ultra commercial device (Rx Solutions, Chavanod, France). The scanner features a Hamamatsu reflection target microfocus X-ray source L10801. The tube was operated at 90kVp, with 110µA, resulting in the smallest possible tube spot size at 5µm. The detector used in this study is a 1880 × 1494 pixel array of flat panel with a physical pixel size of 127µm and 16-bit dynamic range.

The scintillator is a 700µm thick columnar CsI. The exposure time was set to 0.5s to utilize the full dynamic range of the detector, and for each projection, 30 frames were averaged to increase the signal-to-noise-ratio. Total number of projections over 360 degrees per scan is 1440, resulting in 6 hours of theoretical scan time.

The ngTMA blocks were placed vertically on the scanner to maximize the contrast-to-noise-ratio and the scan geometry was designed to maximize the photon economy at the desired voxel size rather than edge enhancement^23^. This compromise was made due to hardware limits and time constraints. The SDD was 667.35mm and the SOD was 26.27mm, resulting in a voxel size of 5.00µm and an effective area coverage of 9.4mm by 7.5mm.

Given the 600µm diameter of each punch and the spacing between the punches, during each scan approximately an array of 7 by 5 punches could be scanned. Therefore, each ngTMA block is scanned in multiple individual scans. Then these scans are stitched together to form the complete array. Consequently, depending on the number of punches in an array, and the spacing between the punches, the net scan time of the ngTMAs can vary.

Then, each individual scan is reconstructed using the commercial reconstruction software, XAct (RX Solutions, Chavanod, France). Where a set of trivial image processing step are applied, first a ring filter correction is applied, followed by a Paganin filter with a $\mathrm {0.25f_{Ny}}$ cut of frequency at $\mathrm {-6dB}$^49^. Then a standard filter back projection algorithm^50^, is applied to reconstruct the volume. Which is then mapped to a fixed unified gray value contrast window for all the individual scans.

### Image processing

#### Segmentation

The segmentation process begins by computing a mean intensity projection along the z-axis, transforming the 3D ngTMA volumes into a single 2D image. To reduce noise sensitivity inherent to the subsequent steps, a Gaussian smoothing filter with a kernel size of 5 pixels is applied. Next, Hue’s circle transform from OpenCV is utilized on the smoothed image, using a predefined circle radius of 65 pixels. Detected circles are aligned against a reference grid generated based on the image dimensions and known punches layout. Each detected circle is assigned a gray value linked to specific grid coordinates.

Following circle detection, the circles are duplicated along the depth, creating cylindrical volumes encapsulating each punch. Regions corresponding to air inclusions (identified by zero gray value pixels from the embedding artifacts) are excluded from these cylinders. These exclusion zones are then expanded through morphological dilation using a spherical kernel of 2 pixels, effectively removing embedding artifacts and ensuring their artifacts do not propagate by the downstream image processing steps into the feature extraction process.

#### Filter classes

In the Pyradiomics library, if the filter class is activated, it is calculated on the original image and then rescaled^51^. However, in the case of ngTMAs, it does not work properly. In this scenario, the air embedding artifacts give rise to false signals that tend to dominate in some filter classes (e. g. gradient filter), because of the abrupt change in the gray value at the air inclusions. These dominating signals corrupt the subsequent normalization process, and considering the image discretization that comes naturally in radiomics, all the gray values collapse in one or two bins. Consequently, we need to apply the filters and normalize them manually and feed them as original images to the library. We applied the following filter classes using ”pyradiomics.imageoperations”. **(i)** Laplacian of Gaussian ($\sigma =10\mu m$), **(ii)** wavelet (start_level=0, level=1, wavelet=”haar”), except HHH, **(iii)** logarithm, **(iv)** square Root, **(v)** gradient, **(vi)** exponential, **(vii)** square.

The directional channels in the wavelet decomposition are not meaningful due to the isotropic nature of both tissues and voxel sizes. As a result, automatically only one direction is retained for each feature across the three directional channels after redundancy reduction (see section “Redundancy reduction”). In the SHAP analysis described in section “Classifier evaluation”, any directional information from wavelet channels should be disregarded. For instance, wavelet-LLH, HLL, and LHL are considered equivalent.

#### Normalization

Based on the visual observation of the filtered images, a histogram window is selected such that it does not saturate the upper histogram and does not cut off gray values at the lower end of the histogram. For each filter class, this window is unified and does not change from one scan to another. Normalized images are then discretized to 8 gray levels to avoid sparse matrices.

### Feature extraction

Feature extraction is done using PyRadiomics library^51^ with 8 gray levels. the activated feature classes are **(i)** First order, **(ii)** gray level co-occurrence matrix (GLCM), **(iii)** gray level run length matrix (GLRLM), **(iv)** gray level dependencies matrix (GLDM), **(v)** gray level size zone matrix (GLSZM), **(vi)** neighbouring gray tone difference matrix (NGTDM). Shape features are not extracted since the shape of all the cores is a cylinder. This feature class setting produces 93 features per filter class, resulting in 1302 features.

### Feature Processing

Many of 1302 features are not robust to variations in the imaging protocol, and many of them are rudimentary. Last but not least, the variations in the paraffin properties and its thickness result in batch bias. Therefore, a set of feature processing steps are necessary before using the embeddings for classification tasks. As an initial step, features with zero variance across the dataset are removed, since they carry no discriminative information.

#### Non-reproducible features

While radiomics features provide valuable diagnostic signals, their interpretability and generalizability across cohorts and scanner platforms remain active areas of investigation^52^. To ensure internal robustness and identify stable features for downstream analysis, we evaluated the intraclass correlation coefficient (ICC) between two distinct micro-CT scans of a single ngTMA block containing 193 punches. The first scan utilized previously described standard parameters (as detailed in Section "X-ray micro-CT"), while the second scan involved positioning the ngTMA block horizontally, significantly altering imaging artifacts and quality metrics. Apart from the mounting mode of the sample, scanning parameters were selected such that they are widely different. This ICC analysis serves as an internal technical robustness step for feature stability assessment within this dataset.

The first scan is from the dataset acquired through the protocol described in section "X-ray micro-CT", the second scan featured modified geometry: source-to-detector distance (SDD) of 856.14 mm and source-to-object distance (SOD) of 33.72 mm, resulting in a voxel size of 5.00 µm. The exposure time per projection was 0.67 s, with 40-frame averaging across 2912 projections over a full 360-degree rotation. Additionally, we virtually expanded detector coverage by horizontally offsetting and stitching two projections per angle, effectively doubling detector width and achieving an expanded field of view. This method resulted in a theoretical total scan duration of approximately 43 hours and 8 minutes for a full ngTMA block.

Images from both scans underwent identical reconstruction and embedding procedures, consistent with the methodologies outlined in Sections “Image processing” and section “Feature extraction”, to ensure validity for ICC analysis. Next, intraclass correlation was calculated between the two sets of features, using the ”intraclass_cor” function from the Penguin library in Python. Features with an ICC smaller than 0.75 and a p-value smaller than 0.05 were discarded as non-reproducible.

#### Batch correction

Batch effects in ngTMA radiomics datasets can arise from variations in chemical properties of paraffin wax, subtle differences in scanning geometry, or hardware instabilities during image acquisition. These batch-related variations introduce non-biological discrepancies among extracted radiomics features.

Prior to correction, clustering of the six ngTMA batches is clearly observed in the UMAP visualization of the radiomics feature space, as demonstrated in Fig. S3(a). The six batches correspond to the four ngTMA blocks, two of which contained two separate grids each and were therefore treated as two independent batches. Batch correction was performed on the full dataset before cross-validation to ensure consistent batch effect removal across the entire cohort. First, a sign-preserving log-transform is applied to the features to reduce skewness and stabilize variance. Batch correction is then performed using ComBat^53^, from the pyCombat package^54^, which effectively mitigates the batch-related clustering, yielding biologically-driven feature distribution as illustrated in Fig. S3(b). ComBat was chosen due to its previously demonstrated effectiveness in radiomics applications^55^, providing robust correction of batch effects.

#### Redundancy reduction

Following batch correction, redundancy reduction was also performed on the full dataset before cross-validation to ensure feature stability. Feature redundancy is addressed by calculating Pearson correlation coefficients between all possible feature pairs using the SciPy library in Python. Feature pairs exhibiting a correlation coefficient greater than 0.75 and a p-value less than 0.05 are identified as highly redundant. From each redundant pair, one feature is randomly selected and discarded iteratively until all redundancy is resolved. We ended up with 54 features that are robust and informative. The resulting feature distribution by filter and feature class is given in table 2.

**Table 2:** Distribution of the 54 retained features by filter and feature class.

**Filter Class**	**# Features**	**Feature Class**	**# Features**
Gradient	1	First-order	16
Log-Sigma-10*μ*m	13	GLCM	12
Logarithm	4	GLDM	2
Original	1	GLRLM	9
Wavelet	35	GLSZM	12
		NGTDM	3

### Classifier

#### Architecture and training

We trained a feed-forward neural network (Multilayer Perceptron, MLP) composed of three fully connected hidden layers followed by an output layer. We chose MLP because of its flexibility and its ability to capture non-linear relationships between features. To justify this choice, we benchmarked four model families: logistic regression (LR), support vector machine with RBF kernel (SVM), random forest (RF), XGBoost (XGB), and MLP. Mean AUC across 5-fold cross-validation for the three main tasks is reported in table S3. MLP matched or outperformed all baselines, with the most pronounced advantage on the PTC vs FTN task, where LR and SVM showed substantially lower and more variable performance. MLP was retained as the primary classifier for its consistent performance across tasks and its compatibility with SHAP-based feature attribution. The input to the model is a 54-dimensional radiomics embedding. Each hidden layer was followed by a ReLU activation and a dropout layer to mitigate overfitting, with the three hidden layer widths set per task (table S1). A final fully connected output layer mapped the last hidden representation to the target classes. We trained the model using an adam optimiser by minimizing a standard cross-entropy loss with weight decay to regularize the model parameters. We used a learning rate scheduler for stable convergence. This architecture captures non-linear relationships among the radiomics features while remaining compact and straightforward to optimize.

The following hyperparameters were kept constant across all classification tasks: learning rate (0.001), weight decay (0.001), and learning rate scheduler gamma (0.9). Varying hyperparameters for each classifier are given in table S1.

#### Classifier evaluation

All classifiers were evaluated using stratified patient-level 5-fold cross-validation. Within each fold, input features were standardized to zero mean and unit variance using statistics computed from the training partition only, so that no validation information influenced the scaling. Performance metrics, including loss, accuracy-based scores, and area under the ROC AUC, were computed for both training and validation sets and are summarized in table S2. The accuracy-based metrics were calculated at the 0.5 probability threshold, except for the TERT mutation status classifier, where the threshold for positive class probability was lowered to 0.2. The probability distribution plots show the aggregated predicted class probabilities from all validation folds in the cross-validation analysis. Ninety-five percent confidence intervals for AUC and AUPRC were computed using 2000-iteration bootstrap resampling of the aggregated validation predictions. These distributions are visualised using Gaussian kernel density estimation (KDE) applied to the aggregated fold outputs.

For 2D visualization of the dataset, we applied Uniform Manifold Approximation and Projection (UMAP)^56^ to project the high-dimensional feature space into two dimensions. Probability distributions are reported as aggregated outputs across the cross-validation folds. To explain the model’s predictions, we used SHAP (SHapley Additive exPlanations) with the KernelExplainer method^39^. We defined a custom prediction function that applies the same scaling and model used during training. A set of 200 samples from the training data was used as the background for computing SHAP values. This allowed us to measure how much each feature contributed to the model’s output for each sample. SHAP attributes contributions to the model’s output and does not by itself establish a causal biological mechanism; the textural interpretations presented in the Results are therefore descriptive associations between imaging phenotype and model behavior, not demonstrated causal links.

### Immunohistochemistry

Immunohistochemical staining was performed on FFPE tissue sections. For BRAF V600E detection, the VE1 clone (Ventana Roche, 790–4855, RTU) was used. Antigen retrieval was carried out with Tris-EDTA buffer (pH 9.0) for 72 minutes at 99°C. Staining was performed using the Benchmark Ultra automated staining system (Ventana Medical Systems). For RAS Q61R detection, the SP174 clone (Abcam, ab227658, dilution 1:50) was applied. Antigen retrieval was performed with Tris-EDTA buffer (pH 9.0) for 40 minutes at 95°C. Staining was conducted on the Leica BOND III automated platform (Leica Biosystems). Notably, the antibody used detects Q61R mutations in HRAS, KRAS, and NRAS, while in PTC, NRAS and HRAS mutations are the most frequently observed.

### TERT mutational analysis

TERT promoter mutation analysis was performed as previously described^57^. Briefly, DNA was extracted from FFPE tissue using the DNeasy Blood and Tissue Kit (Qiagen, Hombrechtikon, Switzerland). A DNA fragment encompassing both mutational hotspot regions of the TERT promoter was amplified using the following primer pair: 5’-CACCCGTCCTGCCCCTTCACCTT-3’,

and 5’-GGCTTCCCACGTGCGCAGCAGGA-3’. The PCR products were subsequently subjected to Sanger sequencing.

## Supplementary Information

Supplementary Information.

## Data Availability

The ngTMA images will be made available following completion of the peer review process. Radiomics embeddings (to be added post-review), corresponding labels, and all code used for image processing, feature extraction, and classification are publicly accessible at: [GitHub Repository.](https:/github.com/kiataj/ngTMAs-radiomics).
