# Supplementary material for: 3D radiomics profiling of thyroid tumors using micro-CT
Source: Sci Rep. 2026 Jun 17;16:27720. doi: 10.1038/s41598-026-57746-1 (PMC13542263; doi:10.1038/s41598-026-57746-1)
Supplement: Supplementary file 1 [file 41598_2026_57746_MOESM1_ESM.pdf]

Supplementary materials

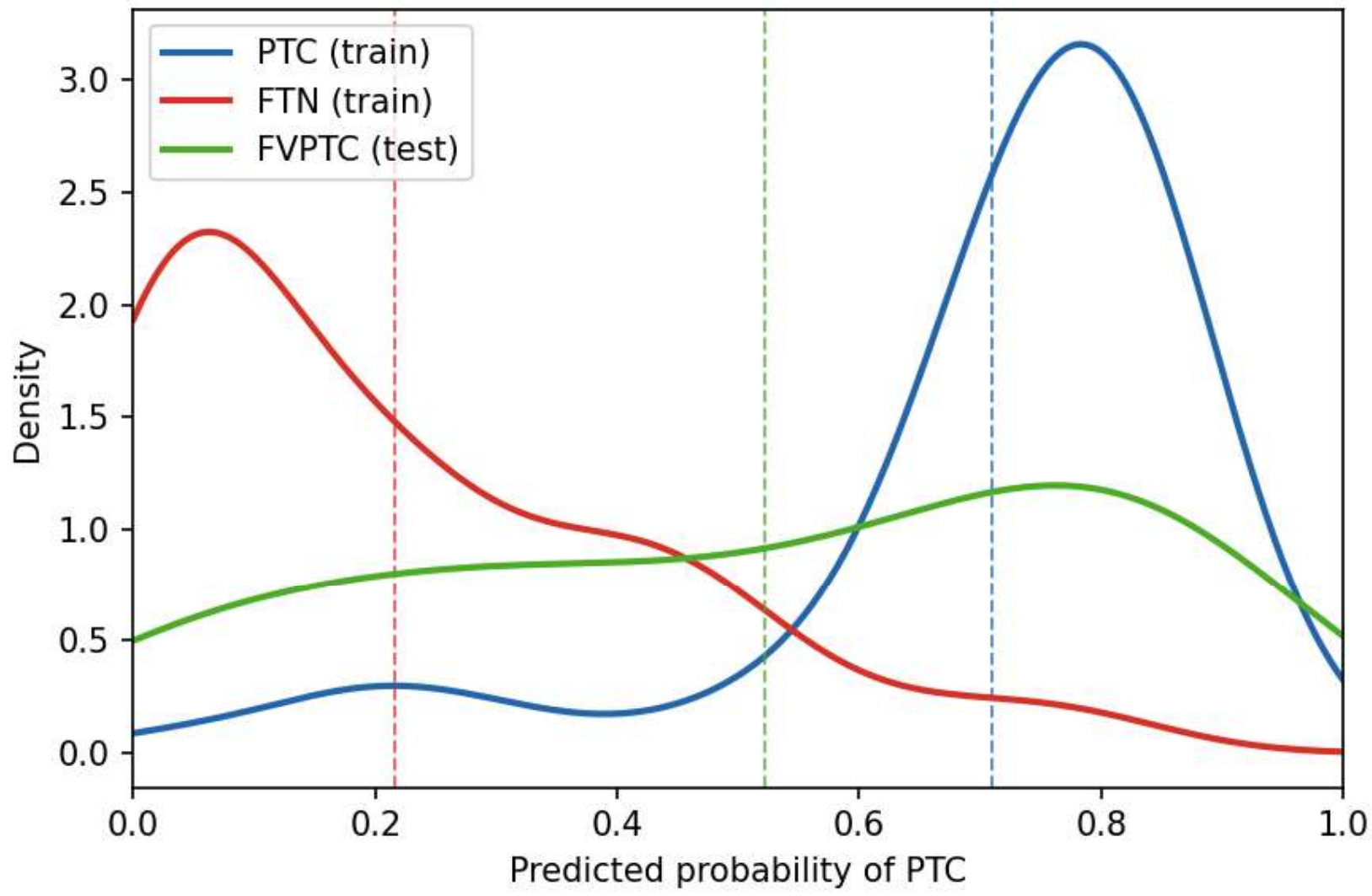

Figure S1: Kernel density estimates of the predicted probability of PTC for PTC training samples (n=97, blue), FTN training samples (n=116, red), and FVPTC samples (n=47, green) applied as an out-of-distribution test to the trained PTC vs. FTN classifier. Dashed vertical lines mark the mean of each distribution. The mean predicted probability of PTC for FVPTC is 0.52 (median 0.58). JSD(FVPTC, PTC) = 0.227 is lower than JSD(FVPTC, FTN) = 0.324, confirming that FVPTC is distributionally closer to PTC.

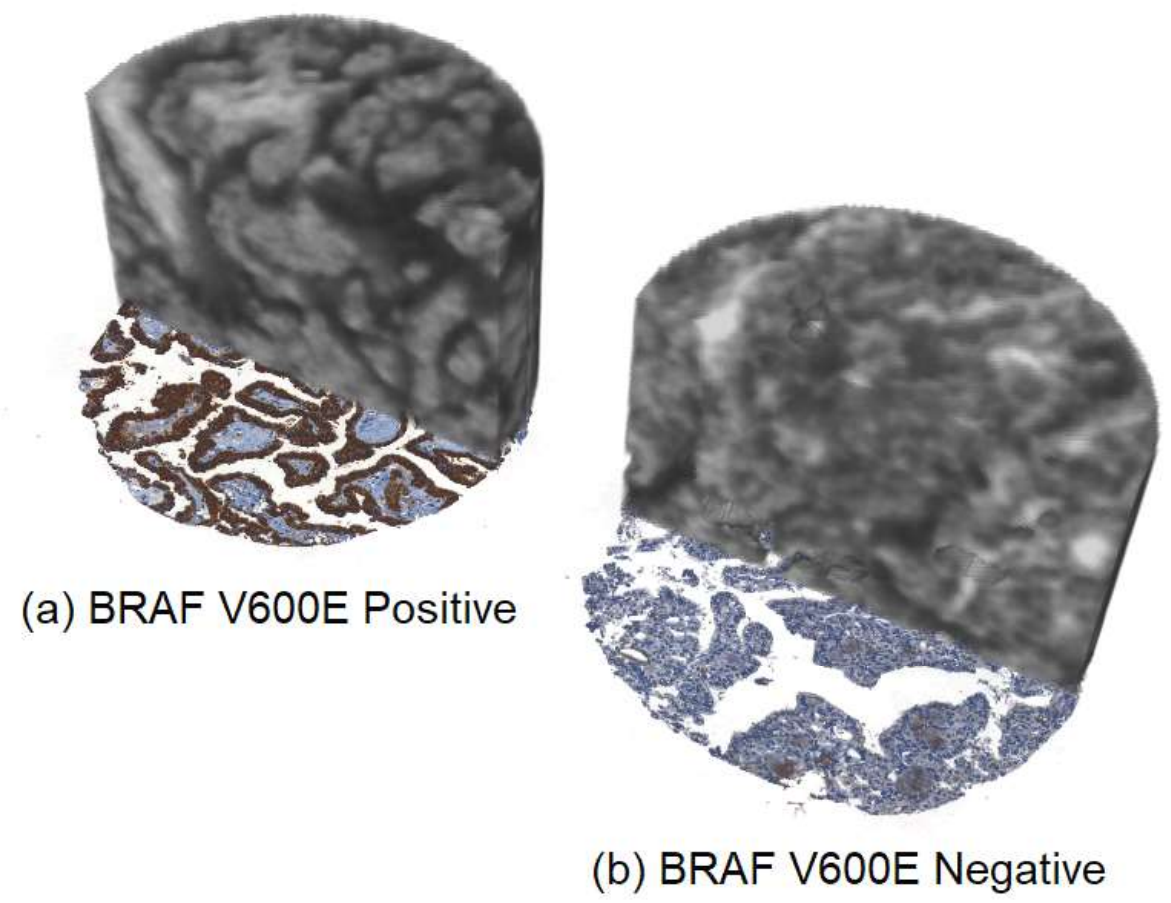

Figure S2: Two punches, (a) BRAF V600E positive, and (b) BRAF V600E negative, their corresponding BRAF V600E immunohistochemistry image is shown at the bottom of punch. Each punch is 600μm in diameter. The classifier predicted a BRAF V600E mutation probability of 84% for (a) and 36% for (b).

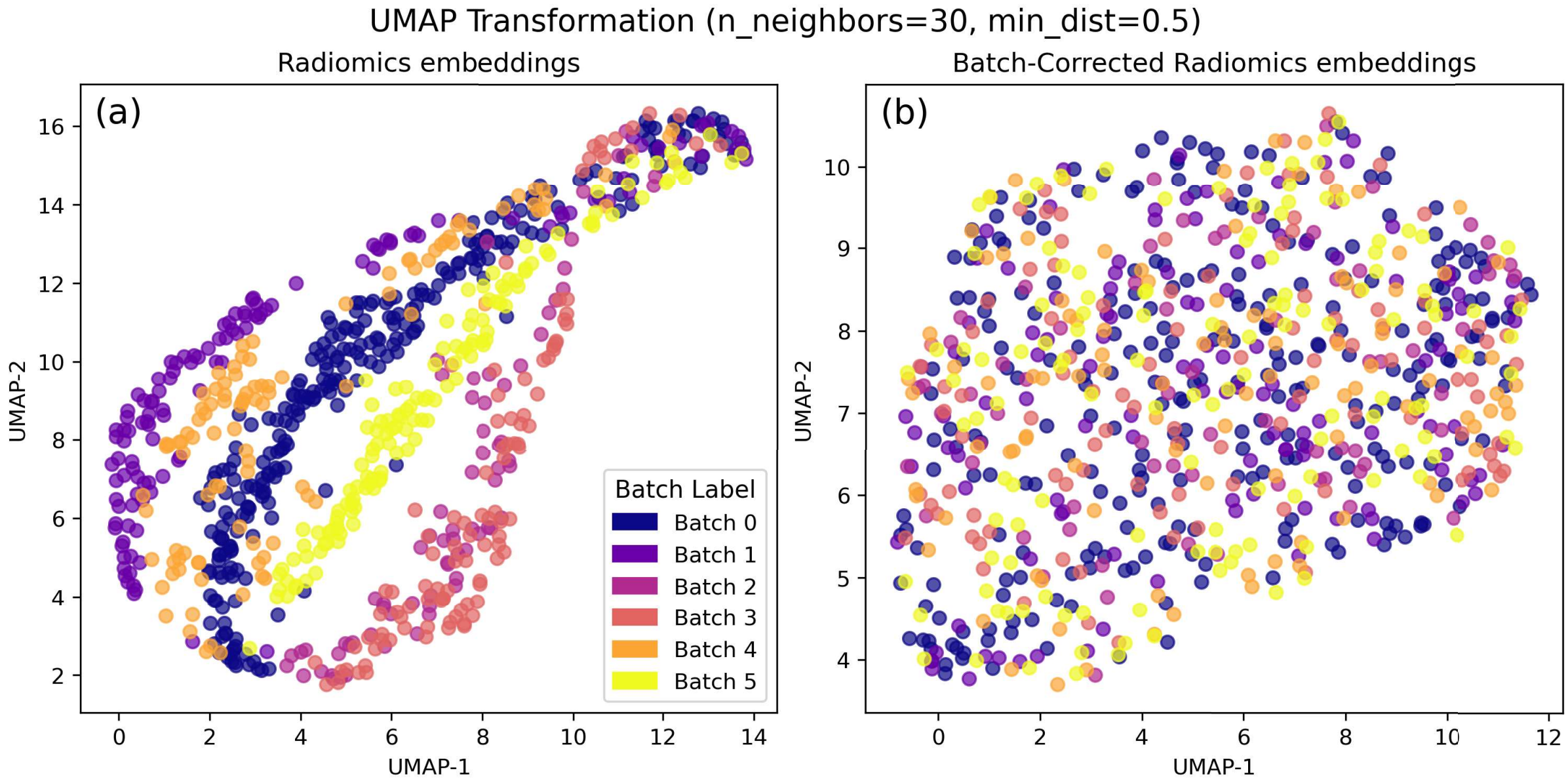

Figure S3: UMAP of dataset (a) before and (b) after batch correction. The projection includes all 800 punch samples across six batches from four ngTMA blocks.

Table S1: Classifier hyperparameters that vary across classification tasks. Shared parameters are listed in the section 4.6.1.

Hyperparameter	Tissue type	Tumor type	BRAF V600E	TERT	RAS Q61R	Relapse
Hidden Dim	(256,128,256)	(256,128,256)	(256,128,256)	64	128	128
Num Epochs	100	100	100	2	4	5
Class weights	[1, 1]	[1, 1]	[1, 2]	[1, 2]	[1, 2]	[1, 1]
Batch Size	16	32	16	4	16	16
Dropout rate	0.6	0.5	0.5	0.7	0.3	0.3

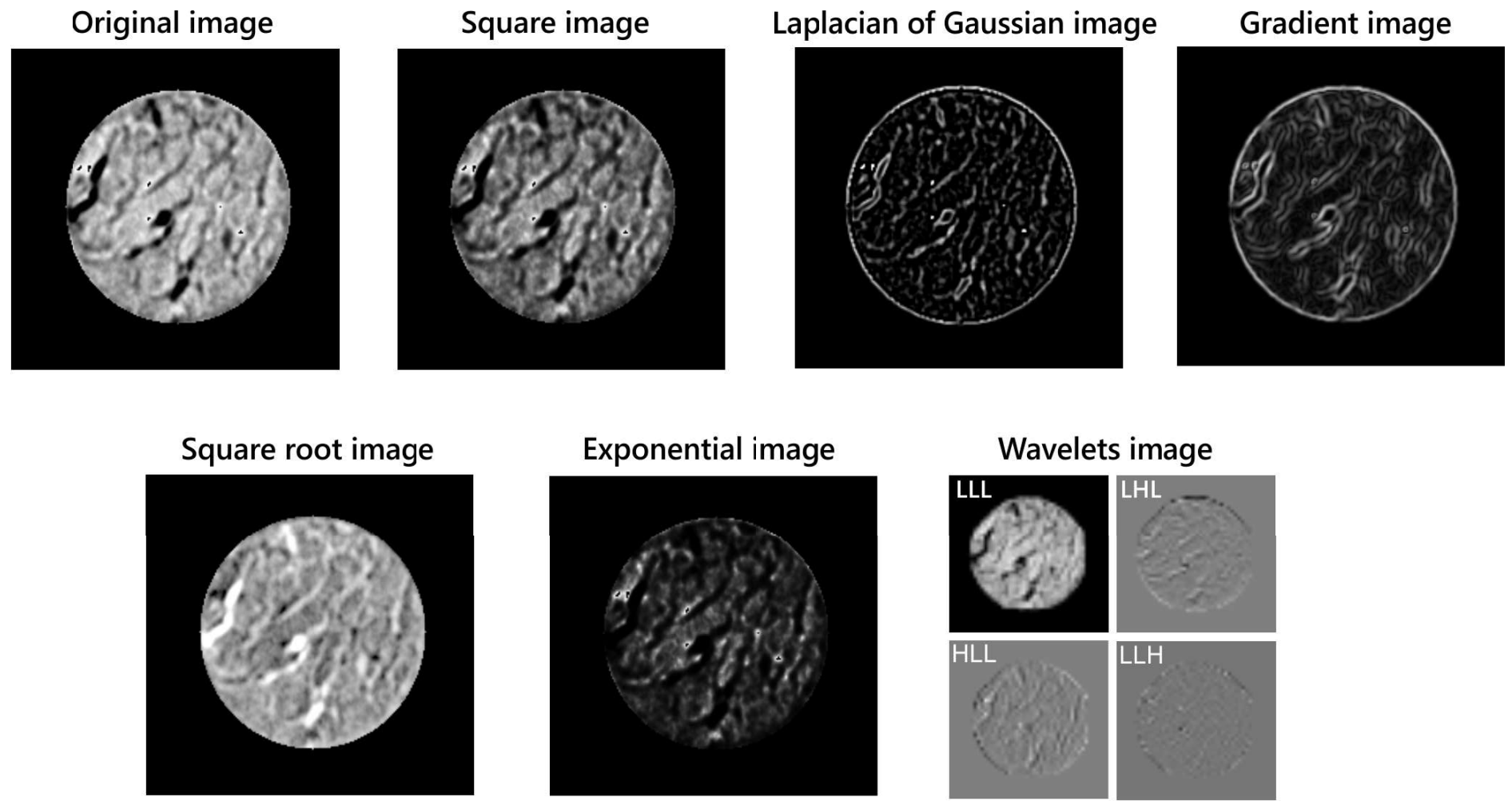

Figure S4: The images demonstrate various filter classes used in radiomics feature extraction. Each image highlights a different aspect of the tissue.

Table S2: 5-fold cross validation training and validation metrics for each classification task. The TERT promoter analysis is reported separately as a Mann–Whitney U test (table 1); AUPRC was not computed for the RAS Q61R and relapse classifiers.

Task	Dataset	Training					Validation				
		AUROC	AUPRC	Precision	Recall	F1	AUROC	AUPRC	Precision	Recall	F1
Tissue type	Non-neoplastic: 336<br>Neoplastic: 405	0.93 ± 0.01	0.94 ± 0.01	0.85 ± 0.01	0.85 ± 0.01	0.85 ± 0.01	0.85 ± 0.04	0.85 ± 0.06	0.77 ± 0.03	0.77 ± 0.03	0.77 ± 0.03
Tumor type	PTC: 97<br>FTN: 116	0.97 ± 0.01	0.97 ± 0.01	0.93 ± 0.02	0.93 ± 0.02	0.93 ± 0.02	0.82 ± 0.05	0.82 ± 0.03	0.75 ± 0.07	0.74 ± 0.07	0.74 ± 0.07
BRAF V600E	Negative: 159<br>Positive: 80	0.96 ± 0.02	0.93 ± 0.03	0.91 ± 0.03	0.90 ± 0.03	0.91 ± 0.03	0.74 ± 0.06	0.59 ± 0.11	0.70 ± 0.04	0.69 ± 0.05	0.69 ± 0.04
RAS Q61R	Negative: 308<br>Positive: 46	0.85 ± 0.02	N/A	0.89 ± 0.01	0.53 ± 0.04	0.59 ± 0.04	0.54 ± 0.09	N/A	0.79 ± 0.06	0.45 ± 0.09	0.51 ± 0.09
Relapse	Negative: 128<br>Positive: 95	0.80 ± 0.01	N/A	0.72 ± 0.02	0.72 ± 0.02	0.70 ± 0.02	0.59 ± 0.04	N/A	0.59 ± 0.07	0.59 ± 0.06	0.57 ± 0.08

Table S3: Mean validation AUC $\pm$ SD across 5 cross-validation folds for all evaluated model families. MLP was selected as the primary classifier.

**Model**	**Tissue type**	**PTC vs FTN**	**BRAF V600E**
MLP	0.848 ± 0.035	0.816 ± 0.046	0.742 ± 0.062
LR	0.835 ± 0.028	0.703 ± 0.125	0.730 ± 0.080
SVM (RBF)	0.858 ± 0.031	0.736 ± 0.081	0.739 ± 0.046
RF	0.841 ± 0.024	0.771 ± 0.067	0.759 ± 0.038
XGBoost	0.851 ± 0.021	0.807 ± 0.048	0.770 ± 0.030
